# Systematic In Vivo Analysis of the Intrinsic Determinants of Amyloid β Pathogenicity

**DOI:** 10.1371/journal.pbio.0050290

**Published:** 2007-10-30

**Authors:** Leila M Luheshi, Gian Gaetano Tartaglia, Ann-Christin Brorsson, Amol P Pawar, Ian E Watson, Fabrizio Chiti, Michele Vendruscolo, David A Lomas, Christopher M Dobson, Damian C Crowther

**Affiliations:** 1 Department of Chemistry, University of Cambridge, Cambridge, United Kingdom; 2 Department of Biochemistry, University of Cambridge, Cambridge, United Kingdom; 3 Department of Genetics, University of Cambridge, Cambridge, United Kingdom; 4 Dipartimento di Scienze Biochimiche, Università degli Studi di Firenze, Firenze, Italy; 5 Department of Medicine, University of Cambridge, Cambridge, United Kingdom; 6 Cambridge Institute for Medical Research, Cambridge, United Kingdom; University of California San Francisco, United States of America

## Abstract

Protein aggregation into amyloid fibrils and protofibrillar aggregates is associated with a number of the most common neurodegenerative diseases. We have established, using a computational approach, that knowledge of the primary sequences of proteins is sufficient to predict their in vitro aggregation propensities. Here we demonstrate, using rational mutagenesis of the Aβ_42_ peptide based on such computational predictions of aggregation propensity, the existence of a strong correlation between the propensity of Aβ_42_ to form protofibrils and its effect on neuronal dysfunction and degeneration in a *Drosophila* model of Alzheimer disease. Our findings provide a quantitative description of the molecular basis for the pathogenicity of Aβ and link directly and systematically the intrinsic properties of biomolecules, predicted in silico and confirmed in vitro, to pathogenic events taking place in a living organism.

## Author Summary

A wide range of diseases, including diabetes and common brain diseases of old age, are characterised by the deposition of protein in the affected tissues. Alzheimer disease, the most common neurodegenerative disorder, is caused by the aggregation and deposition of a peptide called Aβ in the brain. We have previously developed a computational procedure that predicts a particular peptide or protein's speed of aggregation in the test tube. Our goal was to test whether the speed of aggregate formation that we observe in the test tube is directly linked to the brain toxicity that we see in our fruit fly model of Alzheimer disease. We made flies that produce each of 17 variant forms of Aβ and show that the toxicity of each variant is closely linked to the tendency of each variant to form small soluble aggregates. Our computational procedure has previously been shown to be applicable to a wide range of different proteins and diseases, and so this demonstration that it can predict toxicity in an animal model system has implications for many areas of disease-related research.

## Abstract 3

A systematic analysis of Alzheimer disease amyloid β peptide variants in*Drosophila* brain demonstrates that their predicted propensity to form protofibrillar aggregates correlates best with toxicity.

## Introduction

A wide range of proteins has been found to convert into extracellular amyloid fibrils, or amyloid-like intracellular inclusions, under physiological conditions [1,2]. Such proteins have largely been identified through their association with disease, although a number have been found to have beneficial physiological functions in organisms including, amongst others, bacteria [3], yeast [4], and humans [5]. Indeed, the ability to aggregate and assemble into amyloid-like fibrils has emerged as a common, and perhaps fundamental, property of polypeptide chains [1,6,7]. This discovery has stimulated extensive biophysical and mutational analysis of the underlying molecular determinants of amyloid fibril formation. These studies have resulted in the derivation of general models, based on physicochemical parameters, that both rationalise and predict the propensity of proteins to convert from their soluble forms into intractable amyloid aggregates in vitro [8–10].

The misfolding and aggregation of proteins in vivo, however, differ from similar processes taking place under in vitro experimental conditions, in that they occur in complex cellular environments containing a host of factors that are known to modulate protein aggregation and protect against any subsequent toxicity [11]. This difference between in vitro and in vivo experimental conditions represents a significant barrier to the development of a molecular understanding of protein aggregation in living systems and its consequences for disease. In this paper we describe the results of an approach designed to bridge this divide by expressing a range of mutational variants of Aβ_42_ in a *Drosophila* model of Alzheimer disease [12] and correlating their influence on the longevity and behaviour of the flies with their underlying physicochemical characteristics.

## Results/Discussion

**Figure 1:**
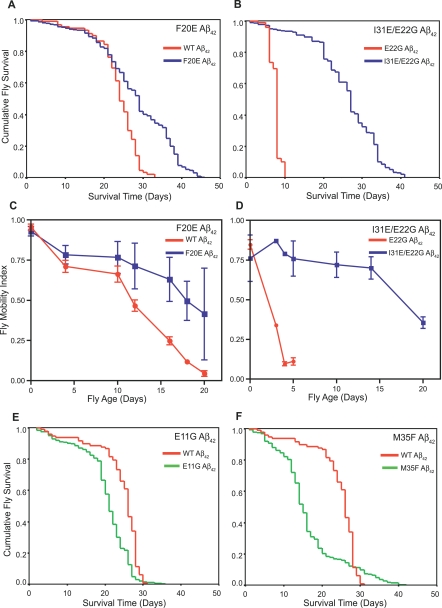
Correlation between Predicted Aggregation Propensity and In Vivo Effects of Aβ_42_ Mutants (A) Flies expressing F20E Aβ_42_ (blue line) live significantly longer (median survival 29 ± 1 d, *n =* 400, *p <* 0.0001) than flies expressing WT Aβ_42_ (red line) (median survival 24 ± 1 d, *n =* 100). (B) Flies expressing I31E/E22G Aβ_42_ (blue line) show a dramatic increase in longevity (median survival = 27 ± 1 d, *n =* 600, *p <* 0.0001) compared to flies expressing E22G Aβ_42_ (red line) (median survival = 8 ± 1 d, *n =* 100). (C) Flies expressing the F20E Aβ_42_ peptide (blue squares) have significantly improved locomotor ability (*p <* 0.001, *n =* 90 observations per line per time point) compared with flies expressing the WT Aβ_42_ peptide (red circles). (D) Flies expressing the I31E/E22G Aβ_42_ peptide (blue squares) have significantly improved locomotor ability (*p* < 0.001, *n* = 90 observations per line per time point) compared to flies expressing E22G Aβ42 (red circles). (E) Flies expressing E11G Aβ_42_ (green line) die significantly quicker (median survival 21 ± 1, *n =* 500, *p <* 0.0001) than those expressing WT Aβ_42_ (red line) (median survival 26 ± 1 d, *n =* 100). (F) Flies expressing M35F Aβ_42_ (green line) die significantly more quickly (median survival = 15 ± 1, *n =* 500, *p <* 0.0001) than those expressing WT Aβ_42_ (red line) (median survival 26 ± 1 d, *n =* 100). Larger values of *S*_tox_ indicate higher toxicity

The expression of the Aβ_42_ peptide (coupled to a secretion signal peptide) in the central nervous system of Drosophila melanogaster results in both intracellular and extracellular deposition of Aβ_42_, along with neuronal dysfunction, revealed by abnormal locomotor behaviour and reduced longevity [12–14]. Learning and memory deficits are also observed in flies expressing Aβ_42_ and to a lesser extent in those expressing Aβ_40_. Importantly, the severity of the cognitive deficits is closely correlated with the magnitude of the locomotor and longevity phenotypes [14]. Our system, as with other recently developed invertebrate models of neurodegenerative disease, therefore produces clear, quantitative phenotypes that allow rapid and statistically robust assessments of the effects of mutations [15,16]. Using an algorithm described previously [8,10] we computed the intrinsic aggregation propensities (*Z*_agg_) of all 798 possible single point mutations of the Aβ_42_ peptide and also of the more toxic E22G Aβ_42_ peptide. A total of 17 mutational variants, with a wide range of aggregation propensities (Table 1), were then expressed throughout the central nervous system of *Drosophila melanogaster,* and their effects were compared to those of wild-type (WT) and E22G Aβ_42_ expression. The longevity of multiple lines of flies (*n* = 4–6 independent lines) for each variant was compared to that of flies expressing the WT or E22G Aβ_42_ peptide. This pooling of data from multiple independent lines for each Aβ_42_ mutant studied serves as a control for the potential variation in expression levels between transgene insertion sites. In addition, the locomotor ability of a representative selection of the Aβ_42_-variant-expressing flies was assessed to provide a measure of the early effects of the peptides on neuronal dysfunction. Examples of the results of this analysis are shown for four of the variants studied (Figure 1).

**Table 1:**
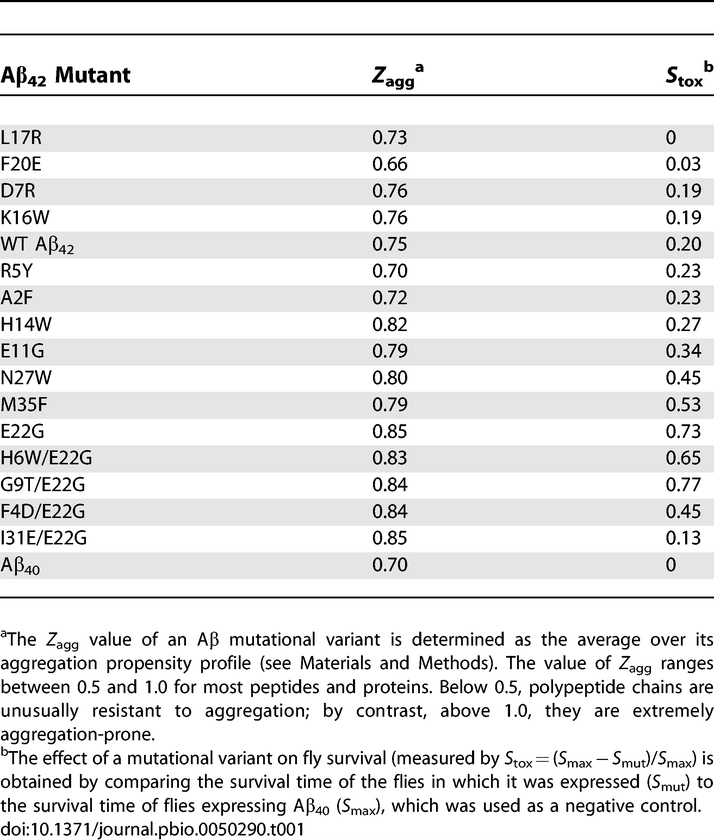
Predicted Aggregation Propensity and In Vivo Toxicity of the Aβ Variants Studied

Flies expressing the WT Aβ_42_ peptide have a median survival of 24 ± 1 d; flies expressing the E22G Aβ_42_ peptide associated with familial Alzheimer disease have a median survival of only 8 ± 1 d. In contrast, some of the peptide variants are less harmful. For example, flies expressing F20E Aβ_42_ have a median survival of 29 ± 1 d (Figure 1A), and flies expressing I31E/E22G Aβ_42_ peptide have a median survival of 27 ± 1 d (Figure 1B), representing substantial increases in longevity compared to WT and E22G Aβ_42_ flies. Furthermore, the longevity of these variants is comparable to that of flies expressing the Aβ_40_ peptide (median survival = 30 ± 1 d; Figure S1A and S1B), which has been previously shown to be non-toxic when expressed both in transgenic flies [12,13] and in transgenic mice [17]. F20E Aβ_42_ and I31E/E22G Aβ_42_ flies also have very significantly improved locomotor ability compared to WT and E22G Aβ_42_ flies (Figure 1C and 1D; Videos S1 and S2) and are comparable in locomotor performance to flies expressing the Aβ_40_ peptide (Figure S1C and S1D). We also analysed a range of Aβ_42_ variants that were more harmful than the WT peptide; for example, flies expressing the E11G or M35F variants of the Aβ_42_ peptide have significantly shorter lifespans than WT Aβ_42_ flies (median survival = 21 ± 1 and 15 ± 1 d, respectively; Figure 1E and 1F).

**Figure 2:**
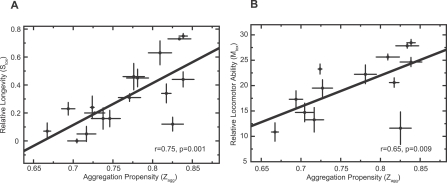
Correlation between In Vivo Toxicity and Aggregation Propensity (*Z*_agg_) (A) There is a significant correlation between the propensities of the Aβ_42_ variants to aggregate (*Z*_agg_) and the relative survival of the flies (*S*_tox_; *r =* 0.75, *p =* 0.001). (B) There is a similarly significant correlation between the propensities of Aβ_42_ variants to aggregate (*Z*_agg_) and the locomotor abilities of the flies (*M*_tox_; *r =* 0.65, *p =* 0.009). In both panels the errors in the in vivo measurements (*y*-axis) are standard errors of the mean arising from the average of the independent lines tested for each variant. The errors in the predictions of aggregation propensity (*Z*_agg_) are also shown (*x*-axis).

**Figure 3:**
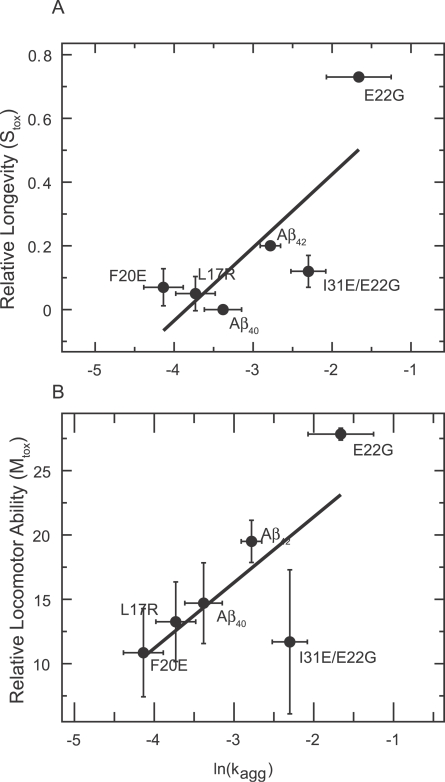
Correlation between Measured Aggregation Rate (*K*_agg_) and both Longevity (*S*_tox_) and Locomotor Performance (*M*_tox_) There is a significant correlation between neuronal dysfunction measured both by longevity (A) (*S*_tox_; *r =* 0.79, *p =* 0.017) and mobility (B) (*M*_tox_; *r =* 0.73, *p =* 0.03) and the rate of aggregation (*K*_agg_) in vitro (measured by thioflavin T fluorescence).

Quantitative analysis of all 17 Aβ variants studied reveals a highly statistically significant correlation between the propensity of a variant to aggregate (*Z*_agg_) and its effect on the survival of the flies (*S*_tox_) (Figure 2A; *r =* 0.75, *p =* 0.001). A significant correlation is also observed when we analyse the relationship between the predicted aggregation propensity (*Z*_agg_) of a representative selection of Aβ variants and their effects on mobility or locomotor performance (*M*_tox_) (Figure 2B; *r =* 0.65, *p =* 0.009). We have also verified that correlations exist between the measured aggregation rates (*K*_agg_) and both *S*_tox_ and *M*_tox_ for a representative selection of the Aβ_42_ variants, as we would expect from our predictions (Figure 3).

Whilst our analysis reveals a significant relationship between the aggregation propensity of Aβ_42_ and its effects on neuronal integrity in vivo, it has also uncovered a small number of variants that do not conform to this trend, most notably the I31E/E22G Aβ_42_ peptide. In order to determine the significance of such divergent behaviour for the origins of Aβ_42_ pathogenicity, we selected one peptide whose effects on the longevity and mobility of the flies is well predicted by its *Z*_agg_ (F20E) and one whose effects did not correlate with its *Z*_agg_ (I31E/E22G) and performed further analysis of their aggregation in vitro and in vivo.

**Figure 4:**
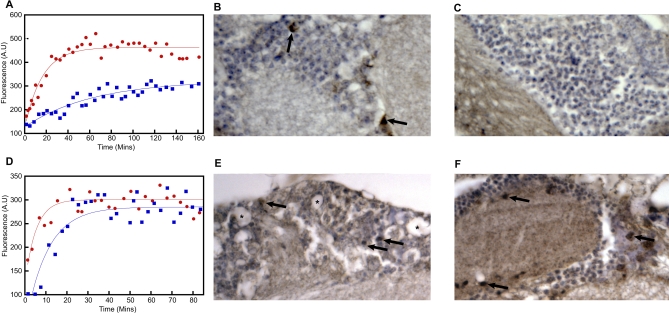
In Vitro and In Vivo Biochemical Analysis of F20E and I31E/E22G Aβ_42_ (A) F20E Aβ_42_ (blue squares) aggregates more slowly than WT Aβ_42_ (red circles), and both were found to have formed well-defined fibrils at the end point of this assay (Figure S3). (B) Immunohistochemistry shows extensive Aβ_42_ deposition (brown staining) in the brain of WT-Aβ_42_--expressing flies at 20 d of age (arrows). (C) In contrast, F20E Aβ_42_ flies show no evidence of Aβ_42_ deposition at 20 d of age. (D) Both E22G (red circles) and I31E/E22G (blue squares) Aβ_42_ aggregate at similar rates as measured by Thioflavin T fluorescence. (E) Aβ_42_ immunohistochemistry shows deposition throughout the cortex in the brain of E22G-Aβ_42_-expressing flies at 8 d of age (arrows). This deposition is also associated with the appearance of vacuoles (asterisks). (F) Flies expressing I31E/E22G Aβ_42_ show extensive deposition of Aβ_42_ throughout their cortex (arrows). In contrast to (E), no evidence of neurodegeneration (vacuolation) is seen.

The F20E mutation is predicted to reduce significantly the propensity of the Aβ_42_ peptide to aggregate (Table 1). Indeed, when we measure the rate of aggregation using thioflavin T fluorescence we find that F20E Aβ_42_ does aggregate significantly more slowly in vitro than the WT Aβ_42_ peptide (*t*_1/2_ = 44 and 11 min, respectively; Figure 4A), in good accord with our predictions.

The in vivo aggregation of the F20E Aβ_42_ peptide is also significantly reduced compared to that of the WT Aβ_42_ peptide. Anti-Aβ_42_ immunohistochemistry using a C-terminal-specific antibody that binds an epitope (Aβ residues 35–42) [18] that does not include the residues being studied here, reveals progressive accumulation of Aβ_42_ in the brains of WT-Aβ_42_-expressing flies from 10 d of age, with extensive deposition being evident by day 20 (Figure 4B). In contrast to this behaviour, flies expressing F20E Aβ_42_ show no signs of Aβ_42_ deposition at day 20 (Figure 4C). Quantitative reverse transcription polymerase chain reaction (qRT-PCR) analysis of Aβ_42_ transcription levels was also carried out on WT Aβ_42_ and two independent lines of F20E Aβ_42_ fly brains to ensure that the reduced deposition and toxicity of the F20E Aβ_42_ peptide was not due to coincidentally lower transcription levels. In fact, the F20E Aβ_42_ transgene was transcribed at slightly higher levels than WT Aβ_42_ (Figure S2) in both lines tested.

That the F20E Aβ_42_ peptide does not form in vivo deposits, despite being able to form amyloid fibrils in vitro (albeit significantly more slowly than WT Aβ_42_) suggests that the F20E mutation reduces the aggregation propensity of Aβ_42_ sufficiently to allow cellular clearance mechanisms such as proteases (e.g., neprilysin) [13] to prevent its accumulation in vivo. We conclude, therefore, that the increased longevity and locomotor performance of F20E Aβ_42_ flies are indeed attributable to a measurable reduction in the aggregation propensity of this peptide in vivo, as predicted by our analysis.

In the case of the I31E/E22G Aβ_42_ variant there appears to be no correlation between its predicted aggregation propensity (which is very similar to that of the highly pathogenic E22G Aβ_42_ peptide; Table 1) and its effects on longevity and locomotor behaviour in the fly (Figure 1B and 1D). However, studies of the I31E/E22G and E22G Aβ_42_ peptides in vitro show that, as predicted by our algorithm, they aggregate at very similar rates (*t*_1/2_ = 7 and 4 min, respectively; Figure 4D). Furthermore, anti-Aβ_42_ immunohistochemistry reveals similar levels of deposition in the brains of both E22G- and I31E/E22G-Aβ_42_-expressing flies at 8 d of age (Figure 4E and 4F) that cannot be accounted for by variations in transcription level as measured by qRT-PCR (Figure S2). Together these observations confirm that our predictions of aggregation propensity are accurate for these peptides in vivo as well as in vitro. To determine the consequences of peptide deposition on the integrity of the brain, we looked for the presence of vacuoles, which are a well-documented sign of neurodegeneration [19]. Despite comparable levels of deposition, the vacuoles seen in the brains of E22G-Aβ_42_-expressing flies are entirely absent from the brains of I31E/E22G-Aβ_42_-expressing flies. In this case, therefore, the relationship between the presence of Aβ_42_ deposits and the functional and anatomical integrity of the brain does not appear to hold.

**Figure 5:**
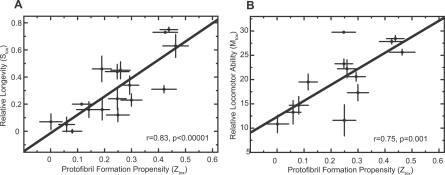
Propensity to Form Protofibrillar Aggregates (*Z*_tox_) as a Predictor of the Effects of Aβ_42_ in Flies (A) *Z*_tox_ predicts more accurately (*r =* 0.83, *p <* 0.00001) than *Z*_agg_ (Figure 2A) the relative longevity (*S*_tox_) of flies expressing different Aβ_42_ variants. (B) *Z*_tox_ predicts more accurately (*r =* 0.75, *p =* 0.001) than *Z*_agg_ (Figure 2B) the relative locomotor ability (*M*_tox_) of flies expressing different Aβ_42_ variants. The errors in *S*_tox_ measurements (*y*-axis) are standard errors of the mean arising from the average of the independent lines of flies tested for each variant. The errors in the predictions of protofibril formation propensity (*Z*_tox_) are also shown (*x*-axis).

This observation is reminiscent of the finding that there are cases in which the presence of Aβ plaques in the brains of elderly humans, and indeed in transgenic mouse models of Alzheimer disease, does not correlate with cognitive ability [20,21]. It has been proposed, in explanation of this finding, that the neuronal dysfunction and degeneration historically attributed to the presence of Aβ amyloid fibrils in the brains of patients with Alzheimer disease may in fact be caused by the concomitant presence of prefibrillar aggregates [22–24]. With this in mind, the unexpected in vivo effects of variants such as the I31E/E22G Aβ_42_ peptide prompted us to develop a second algorithm (see Materials and Methods) by analysing a set of data for which the rates of formation of protofibrils containing β-sheet structure have been reported [8]. This algorithm is able to predict the propensity of other polypeptides to form protofibrils. Whilst there are a few Aβ_42_ variants (including I31E/E22G Aβ_42_) whose global aggregation propensities (Z_agg_) do not correlate well with their in vivo effects on neuronal dysfunction (Figure 2), we find that the predicted propensities of these variants to form protofibrillar aggregates (*Z*_tox_) correlate very strongly with their in vivo effects (*S*_tox_, *r =* 0.83, *p <* 0.00001; *M*_tox_, *r* = 0.75, *p =* 0.001; Figure 5).

We propose, therefore, that the effects of all Aβ_42_ variants in the flies can be directly attributed to their effects on the intrinsic propensities to form deleterious protofibrillar aggregates. It is extremely interesting in this regard that a comparison, using electron microscopy, of the morphology of E22G and I31E/E22G Aβ_42_ aggregates formed under identical conditions reveals the presence of a significant quantity of protofibrils in the former and only well-defined fibrils in the latter (Figure S4). Furthermore, we propose that it is possible to predict accurately in silico the in vivo effects of the Aβ_42_ peptide from a knowledge only of the intrinsic physicochemical properties of its constituent amino acids. We believe that this approach to understanding the determinants of protein misfolding in vivo will be applicable to many other diseases as we have demonstrated previously that the physicochemical parameters that determine the aggregation propensity of Aβ also determine the aggregation behaviour of a wide range of both disease- and non-disease-related proteins [10,25].

It is also remarkable that, despite the fact that the intrinsic aggregation propensities of typical protein sequences vary by at least five orders of magnitude [25], we have been able to achieve profound alterations in the pathogenic effects of Aβ_42_ by increasing or decreasing its propensity to aggregate by less than 15%. This result suggests that proteins implicated in misfolding diseases are likely to be extremely close to the limit of their solubility under normal physiological conditions [26], and consequently the small alterations in their concentration, environment, or sequence, such as occur with genetic mutations [27] or with increasing age [23], are likely to be the fundamental origin of these highly debilitating and increasingly common conditions [28].

In conclusion, we have presented accurate, quantitative measurements of the relationships between the manifestations of neuronal dysfunction in a complex organism, such as locomotor deficits and reduced lifespan, and the fundamental physicochemical factors that determine the propensity of the Aβ_42_ peptide to aggregate into protofibrils. These results provide compelling evidence that, despite the presence within the cell of multiple regulatory mechanisms such as molecular chaperones and degradation systems [29], it is the intrinsic, sequence-dependent propensity of the Aβ_42_ peptide to aggregate to form protofibrillar aggregates that is the primary determinant of its pathological behaviour in living systems.

## Materials and Methods

### Generation of D. melanogaster expressing mutant A**β**_42_ peptides.

Mutant Aβ_42_ expression constructs were produced by site-directed mutagenesis of the WT Aβ_42_ sequence in the pMT vector (Invitrogen) and were subcloned into the pUAST vector. Transgenic *Drosophila* expressing the desired Aβ_42_ variants were generated according to the procedures described by Crowther et al. [12].

### Survival assays.

All survival assays were carried out as described previously [12]. Survival curves were calculated using Kaplan–Meier statistics, and differences between them analysed using the log rank method. All survival times in the text are given as median ± standard error of the median. For previously characterised control lines expressing either WT or E22G Aβ_42_, the survival of one representative line was measured. For each novel mutational variant of Aβ_42_, between four and six independent lines were analysed (*n =* 100 for each line) in order to control for variability in expression levels between individual lines due to the varying location of transgene insertion. The effect of a mutational variant on survival (*S*_tox_) was calculated by comparing the survival time of the flies in which it was expressed (*S*_mut_) to the survival of Aβ_40_-expressing flies (*S*_max_) used as a negative control in the same experiment: *S*_tox_ = (*S*_max_ − *S*_mut_)/*S*_max_.

### Locomotor assays.

The locomotor ability of the flies was assessed in a 45-s negative geotaxis assay. Flies were placed in a plastic 25-ml pipette and knocked to the bottom of the pipette. The number reaching the top of the pipette (above the 25-ml line) and the number remaining at the bottom (below the 2-ml line) after 45 s was measured. The mobility index was calculated as (*n*_top_− *n*_bottom_ + *n*_total_)/2*n*_total_. Two representative lines were tested for each novel mutant Aβ_42_ and one line for each previously characterised control (WT Aβ_42_ and E22G Aβ_42_)_._ Three independent groups of 15 flies each were tested three times at each time point for each line. Differences between genotypes were analysed by ANOVA. The effect of each mutational variant on locomotor performance (*M*_tox_) was calculated by fitting the decline in mobility index over time to a straight line and then estimating the time at which each mutant line of flies had declined to a mobility index of 0.5.

### A**β**_42_ immunohistochemistry analysis.

Immunohistochemistry analysis was performed as described previously [12] on single representative lines for each genotype using the G2–11 anti-Aβ_42_ antibody (The Genetics Company). Representative lines of F20E- and I31E/E22G-Aβ_42_-expressing flies were chosen to have median survivals within 1 d of the combined median survival determined for each genotype.

### Analysing the aggregation propensity of A**β**_42_ mutants.

The propensity to form amyloid aggregates (*Z*_agg_) was calculated using an approach described previously [10]. Briefly, for a given protein, *Z*_agg_ is obtained by averaging the propensities that are above zero in the aggregation profile. All the propensities are normalised into a variable that has an average of zero and a standard deviation that equals one (the normalisation is made using the propensities of a set of random sequences). In a profile there can be residues with a propensity larger than one, but these peaks are usually sparse and their contribution is diluted upon averaging. Consequently, sequences with an overall *Z*_agg_ score larger than one are very rare. In order to calculate the propensity for forming protofibrillar aggregates (*Z*_tox_), we developed a method based on an equation containing the same physicochemical contributions used to calculate the propensity for fibrillar aggregation, but with specific weights determined using a set of experimentally determined protofibrillar aggregation rates for the protein acylphosphatase [30]. A Web server for calculating *Z*_agg_ and *Z*_tox_ is available at http://rd.plos.org/10.1371_journal.pbio.0050290_01.

### A**β** peptide preparation for in vitro kinetic analysis of aggregation.

All peptides were dissolved in trifluoroacetic acid and sonicated for 30 s on ice. The trifluoroacetic acid was removed by lyophilization and the peptides were then dissolved in 1,1,1,3,3,3-hexafluoro-2-propanol and divided into aliquots that were dried by rotary evaporation at room temperature. The amount of peptide in the aliquots was determined by quantitative amino acid analysis.

### In vitro kinetic analysis of A**β**_42_ aggregation.

The peptides were dissolved at a concentration of 30 μM in 50 mM NaH_2_PO_4_ (pH 7.4) and incubated at 29 °C with continuous agitation. At regular time intervals, 5 μl of the peptide solution was removed and added to 100 μl of 20 μM thioflavin T in 50 mM Gly-NaOH (pH 8.5). Fluorescence intensity was measured at 440 nm excitation and 480 nm emission using BMG FLUOstar OPTIMA. The rate of aggregation (*k*) was determined by fitting the plot of fluorescence intensity versus time to a single exponential function *y* = *q* + *A*e^(−*kt*)^ [30], and *t*_1/2_ was calculated using *t*_1/2_ = ln2/*k*.

### qRT-PCR.

Twenty flies expressing each variant of Aβ_42_ were collected at day 0 (i.e., on the day of eclosion) for each transgenic line to be analysed. The flies were then anaesthetised and decapitated, and the heads were collected and snap frozen in liquid N_2_. Total RNA was extracted from the fly heads using the Qiagen RNeasy mini kit with on-column genomic DNA digestion using DNAse 1. The concentration of total RNA purified for each line was measured using a NanoDrop spectrophotometer. One microgram of RNA was then subjected to reverse transcription using the Promega Reverse Transcription System with oligo dT primers. qRT-PCR was performed using a BioRad iCycler and Absolute QPCR SYBR Green Fluorescein Mix (ABgene). Each sample was analysed in triplicate and with both target gene (Aβ_42_) and control gene (RP49) primers in parallel. The primers for the Aβ_42_ PCR were directed to the 5′ end of the signal secretion peptide sequence and the 3′ end of the Aβ coding sequence: forward, GCATTCGTGAATTCATGGCGAGCAAAGT; reverse, TACTTCTAGATCCTCGAGTTACGCAATCAC. The RP49 primers were designed across an intron to avoid amplifying any residual genomic DNA contamination: forward, ATGACCATCCGCCCAGCATCAGG; reverse, ATCTCGCCGCAGTAAACG. Relative expression levels were calculated using the Livak method.

## Supporting Information

Figure S1 The F20E and I31E/E22G Aβ_42_ Variants Rescue the Locomotor and Longevity Phenotype and Are Indistinguishable from Control Flies Expressing Aβ_40_
The Aβ_40_ peptide has been previously demonstrated not to reduce lifespan or locomotor ability compared to non-transgenic flies when expressed in the *Drosophila* central nervous system [12]. The longevity and locomotor ability of a typical line of flies expressing the Aβ_40_ peptide under the control of the *elav* ^c155^ promoter were assessed in parallel with those of the lines of flies expressing F20E and I31E/E22G Aβ_42_ as a negative control for the effects of expressing a less aggregation-prone peptide in the brain of the *Drosophila*.
(A) Flies expressing F20E Aβ_42_ (blue line) did not differ significantly in their longevity from flies expressing Aβ_40_ (red line).
(B) Flies expressing I31E/E22G Aβ_42_ (blue line) have slightly reduced longevity compared to Aβ_40_-expressing flies (red line).
(C and D) Flies expressing F20E or I31E/E22G Aβ_42_ (blue triangles) are indistinguishable in locomotor ability from flies expressing Aβ_40_ (red squares).
(723 KB EPS)

Figure S2 qRT-PCR Analysis of Aβ_42_ Transcription Level for F20E and I31E/E22G Variants of Aβ_42_
The level of Aβ_42_ mRNA present in each of two independent, representative lines of F20E- (F14 and F32) and I31E/E22G- (Isi68 and Isi51) Aβ_42_-expressing flies was compared to the level of Aβ_42_ mRNA in the brains of flies expressing WT and E22G Aβ_42_. All values are relative to the level of WT Aβ_42_ expression and normalised against the level of the housekeeping gene RP49 (see Materials and Methods).
(6263 KB EPS)

Figure S3 Transmission Electron Microscopy of F20E and WT Aβ_42_ Aggregates Samples were taken when the thioflavin T signal had reached a plateau for electron microscopic analysis. Aggregate solutions were placed on formvar-coated nickel grids and stained with uranyl acetate. WT Aβ_42_ (left) and F20E Aβ_42_ (right) show evidence of well-defined fibrils. Scale bar = 200 nm for both panels.
(2.3 MB TIF)

Figure S4 Transmission Electron Microscopy of E22G and I31E/E22G Aβ_42_ Aggregates E22G and I31E/E22G Aβ_42_ were incubated for 24 h at room temperature (25 °C) without shaking in order to minimise disruption of the aggregates and so reveal any differences in morphology between the two samples. Aggregates were prepared as described in Materials and Methods. E22G Aβ_42_ forms both protofibrillar and fibrillar aggregates at this time (left). In stark contrast, I31E/E22G Aβ_42_ forms only well-defined fibrils (right). Scale bar = 500 nm for both panels.
(703 KB TIF)

Video S1 Reducing the Aggregation Propensity of Aβ_42_ Rescues Flies from Locomotor Dysfunction This movie demonstrates the significantly greater locomotor ability of flies expressing F20E or L17R Aβ_42_ compared to that of flies expressing WT Aβ_42_.
(815 KB MOV)

Video S2 Reducing the Aggregation Propensity of E22G Aβ_42_ Rescues Flies from Locomotor Dysfunction This movie demonstrates the significantly greater locomotor ability of flies expressing F4D/E22G or I31E/E22G Aβ_42_ compared to that of flies expressing E22G Aβ_42_.
(1.0 MB MOV)

## Abbreviations

qRT-PCR: quantitative reverse transcription polymerase chain reaction
WT: wild-type
